# Translational medicine in China: improving public health

**DOI:** 10.1186/1479-5876-10-S2-A1

**Published:** 2012-10-17

**Authors:** Daiming Fan

**Affiliations:** 1 Chinese Academy of Engineering, No.2 BingJiaoKou HuTong, Beijing 100088, China

## 

China’s national 12^th^ Five-Year Plan (2011-2015) stated that, by 2015, China should increase the average life expectancy by one year (estimated to reach 74.5 years old) relative to that of 2010. Additionally, China’s new around of health reform was initiated in 2009. Translational medicine, which is moving basic discoveries in the laboratories into human studies to promote new preventions, diagnostics, treatments and cures, is the engine to the health care reform and the proposed life expectancy increase. The potential of translational medicine in China has never been greater.

Starting in 2005, translational research centers began to spontaneously appear in China. Since 2009, more and more centers have been emerged. Currently, translational centers are founded most independently by local cities, universities and hospitals. There’s a lack of policy support and guidance, funding support, resource standardization and sharing. We are still in the exploratory stage. To find better diagnostics and treatments, China’s translational medicine focuses more on innovations and how to push laboratory discoveries to clinical practice. Guided by the concept of “cure a disease before its onset”, China’s translational medicine also puts more effort on public health. As part of translational medicine initiative, China already supported many translational medicine projects during the 11^th^ and 12^th^ Five-Year Plans through National High-Tech R&D Program of China (863 Program), 973 project and National Nature Science Foundation.

As a developing country with 1.3 billion people, China faces many challenges to promote translational medicine and health care reform.

♦ How to combine translational medicine with basic research, clinical medicine and public health?

♦ How does translational medicine better serve China health care reform?

♦ How to further develop traditional Chinese medicine (TCM) through translational medicine?

♦ How to evaluate translational centers and translational medicine programs?

To build a modern health service system and better serve the society, China should list digital health as a priority to develop. Additionally, I would like to suggest that more emphasis should be placed on the establishment of translational medicine-related policies, laws and regulations, resource standardization and sharing. Looking forward, translational medicine is a revolutionary opportunity for China.

